# Application of simple fed-batch technique to high-level secretory production of insulin precursor using *Pichia pastoris *with subsequent purification and conversion to human insulin

**DOI:** 10.1186/1475-2859-9-31

**Published:** 2010-05-12

**Authors:** Chandrasekhar Gurramkonda, Sulena Polez, Natasa Skoko, Ahmad Adnan, Thomas Gäbel, Dipti Chugh, Sathyamangalam Swaminathan, Navin Khanna, Sergio Tisminetzky, Ursula Rinas

**Affiliations:** 1 Helmholtz Centre for Infection Research, Braunschweig, Germany; 2 International Centre for Genetic Engineering & Biotechnology, New Delhi, India; 3 International Centre for Genetic Engineering & Biotechnology, Trieste, Italy; 4 Department of Chemistry, Government College University Lahore, Pakistan; 5 Fraunhofer ITEM, Hannover/Braunschweig, Germany

## Abstract

**Background:**

The prevalence of diabetes is predicted to rise significantly in the coming decades. A recent analysis projects that by the year 2030 there will be ~366 million diabetics around the world, leading to an increased demand for inexpensive insulin to make this life-saving drug also affordable for resource poor countries.

**Results:**

A synthetic insulin precursor (IP)-encoding gene, codon-optimized for expression in *P. pastoris*, was cloned in frame with the *Saccharomyces cerevisiae *α-factor secretory signal and integrated into the genome of *P. pastoris *strain X-33. The strain was grown to high-cell density in a batch procedure using a defined medium with low salt and high glycerol concentrations. Following batch growth, production of IP was carried out at methanol concentrations of 2 g L^-1^, which were kept constant throughout the remaining production phase. This robust feeding strategy led to the secretion of ~3 gram IP per liter of culture broth (corresponding to almost 4 gram IP per liter of cell-free culture supernatant). Using immobilized metal ion affinity chromatography (IMAC) as a novel approach for IP purification, 95% of the secreted product was recovered with a purity of 96% from the clarified culture supernatant. Finally, the purified IP was trypsin digested, transpeptidated, deprotected and further purified leading to ~1.5 g of 99% pure recombinant human insulin per liter of culture broth.

**Conclusions:**

A simple two-phase cultivation process composed of a glycerol batch and a constant methanol fed-batch phase recently developed for the intracellular production of the Hepatitis B surface antigen was adapted to secretory IP production. Compared to the highest previously reported value, this approach resulted in an ~2 fold enhancement of IP production using *Pichia *based expression systems, thus significantly increasing the efficiency of insulin manufacture.

## Background

Diabetes is a progressive disease characterized by hyperglycemia, resulting from defects in insulin secretion, its function, or both. The long-term effects of this disease, for which there is no cure, lead to multiple organ damage and failure [1]. The World Health Organization estimates that ~2.9 million deaths are attributable to diabetes every year [2]. Based on demographic changes, it has been estimated that the number of diabetics around the world, which was ~171 million at the turn of this century, will more than double by the year 2030 [3]. This is likely to be an underestimate given that factors, such as improved life expectancy and obesity contributing to increased prevalence of diabetes, have not been taken into consideration.

Insulin is a 51 amino acid (aa) polypeptide hormone essential for normal glucose homeostasis and is therefore useful in treating diabetes. Insulin contains two polypeptide chains, A (21 aa) and B (30 aa), with three disulfide bonds. Two of these interlink the A- and B-chains, while the third one is an intra A-chain bond [4,5]. Though, initially, insulin was isolated from porcine pancreas, the advent of recombinant DNA technology helped to address the requirement for insulin more effectively. However, commensurate with the projected escalation in the prevalence of diabetes in the coming decades [3], there will be an increasing demand for insulin. To meet this, more cost-effective biotechnological strategies for recombinant insulin production are required.

Insulin was the first recombinant product approved by the FDA for human application [6,7]. The first expression systems were based on the separate expression of the A- and B-chains fused to carrier proteins in two different *E. coli *strains [8,9]. Nowadays, human insulin is produced as recombinant protein, using two major routes. One route involves the production of an insulin precursor in the form of inclusion bodies, using *E. coli *as expression host with subsequent solubilization and refolding procedures [10]. The other route involves the utilization of yeast-based expression systems, leading to the secretion of a soluble insulin precursor (IP) into the culture supernatant [11-13]. Both routes are economically viable. Due to the extensive experience in large-scale cultivation, *Saccharomyces cerevisiae *became the first yeast-based expression system for secretory IP production [11,12]. Though *S. cerevisiae *is still the predominant yeast system for insulin production, several alternative yeast hosts have become available in recent years [14-18]. Of these, the methylotrophic yeast *Pichia pastoris *has emerged as a very useful expression host with superior features [18-21]. In particular, its reliance on integrative vectors, its strong and tightly regulated methanol-inducible alcohol oxidase 1 (*AOX1*) promoter and its capacity to reach very high cell densities by simple cultivation strategies collectively contribute to stable and high level production of recombinant proteins.

Several reports have also demonstrated the utility of *P. pastoris *for secretory IP production [22-30]. The yields reported so far vary over a wide range, with the highest being 1.5 gram IP per liter of culture broth (Table 1). Thus, *P. pastoris *has proven its applicability for insulin production, and, in fact, a comparative study revealed an equal or even better performance of *P. pastoris *compared to *S. cerevisiae *[22].

**Table 1:** Insulin precursor production with *P. pastoris*

Strain (copy#)	MeOH (%)	Induction time (h)	Final biomass **(g L**^**-1**^)	**Product **^**a**^ **(g L**^**-1**^) Culture supernatant	**Product **^**b**^ **(g L**^**-1**^) Culture broth	Mass	**Reference **^**c**^
Mut^+/s ^(6-8)	nd ^d^	87	nd	1.5	nd	nd	[26]
Mut^s ^(nd)	1.0	72	80 to 90	0.22	nd	nd	[25]
Mut^s ^(11)	<0.1	110	89	0.3	nd	MS ^e^	[24]
Mut^s ^(11)	1 to 0.5	136	109	0.25	nd	MS	[23]
Mut^s ^(nd)	nd	72	nd	3.6	1.47	nd	[27]
Mut^+ ^(12)	0.8	96	nd	0.18	nd	nd	[28]
Mut^+ ^(~2) ^f^	0.2	88	59	3.84	3.075	MS	Present study

^a ^Concentrations were determined by reverse phase HPLC from clarified culture supernatant

^b ^Concentrations calculated considering the volumetric portion of biomass in the culture broth

^c ^Only references on secretory insulin precursor production with *P. pastoris *included containing respective quantitative data

^d ^nd; not determined

^e ^MS; mass spectrometry

^f ^Copy number not experimentally determined. Survival at zeocin concentrations up to 0.5 mg mL^-1 ^indicates a copy number of 2 [41].

For secretory IP production using *P. pastoris *as host, the *S. cerevisiae *based α-factor pre-pro leader is most often employed as secretion signal [13]. In general, the insulin B- and A-chains are joined by a short connecting peptide linker, which allows tryptic precursor processing for generation of human insulin [22,25,26,31]. Secretion yields of the single chain insulin precursors can be enhanced by adding a charged synthetic spacer peptide in front of the insulin B-chain segment, which allows more efficient Kex2 endoprotease processing after the α-factor pre-pro leader [13,22,32]. After recovery from the culture broth, IP can be converted enzymatically into human insulin [26,27,29,33,34].

We recently devised a robust cultivation strategy for high-level intracellular production of the Hepatitis B surface antigen (HBsAg), using *P. pastoris *as expression host [35]. In this procedure, cells were first grown to high-cell density in a batch process, using a simple defined medium with low salt and high glycerol concentrations. After batch growth, induction of high-level product formation was achieved by adding methanol to a final concentration of 6 g L^-1 ^and keeping this high concentration for the remainder of the production phase. In the present work, we have adapted this strategy to secretory IP production. Further, we also present a novel purification procedure, based on the metal binding affinity of IP and, finally, describe the conversion of the insulin precursor to human insulin of high purity by enzymatic and chemical processing.

## Results

### Construction of the IP-secreting *P. pastoris *clone

**Figure 1:**
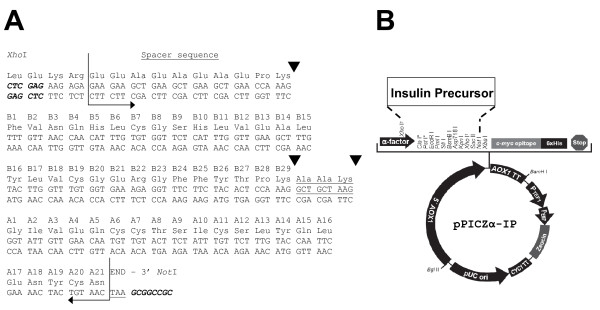
**Construction of the recombinant *P. pastoris *X-33 strain**. (A) The IP gene and polypeptide sequence. The bent arrows indicate the ends of the IP gene and polypeptide. The tripeptide linking the B and A chains is underlined. The nucleotides comprising the 5' and 3' restriction sites are shown in italics. The A and B chain aa residue numbers carry the cognate 'A' and 'B' prefixes, respectively. The black triangles denote the sites of trypsin cleavage. (B) Map of the yeast integrative vector encoding IP. The IP is cloned between the *Xho*I site in the α-factor signal encoding sequence and the *Not*I site in the polylinker.

The IP encoding gene, codon-optimized for expression in *P. pastoris*, was obtained by chemical synthesis. The polypeptide encoded by this gene carries at the N-terminus 4 amino acids corresponding to the carboxy-terminal part of the *S. cerevisiae *α-factor secretory signal (LEKR) which contains the Kex2 cleavage site, followed by a spacer peptide (EEAEAEAEPK) for more efficient Kex2 processing and secretion, followed by the insulin B chain amino acids 1-29, a short connecting linker (AAK), and finally the insulin A chain amino acids 1-21. This gene was fused in-frame with the *S. cerevisiae *α-factor secretory signal in the *Pichia *integrative vector pPICZα to generate the expression plasmid pPICZα-IP (Fig. 1). The IP gene carries its own stop codon and, consequently, does not contain the vector-encoded carboxy-terminal *c-myc *and His_6 _tags. The IP secreted by this vector, following Kex2 cleavage, is predicted to be 63 amino acids long with a molecular mass of ~7 kDa. The plasmid pPICZα-IP was linearized with *Sac*I and electroporated into *P. pastoris *strain X-33 and the resulting transformants selected on zeocin plates, containing progressively increasing concentrations of zeocin (0.1 - 2.0 mg mL^-1^) to identify multicopy clones. The maximum zeocin concentration at which clones could be recovered was 0.5 mg mL^-1^. Several clones that were viable at this zeocin concentration were tested in small scale induction experiments. Extracellular IP concentrations were analyzed by RP-HPLC to identify the best expressing clone. The methanol utilization phenotype of this clone was found to be *Mut*^*+*^, indicating that integration had occurred at the *AOX1 *locus by a single crossover event.

### Production of IP in fed-batch culture

Initial attempts for secretory production of IP were based on the direct application of the fed-batch procedure recently developed for high-level intracellular production of HBsAg with *P. pastoris *[35]. Employing these conditions, cells were first grown in a batch procedure using a simple defined medium with low salt and high glycerol concentrations. After depletion of glycerol, induction of product formation was achieved by adding methanol to a final concentration of 6 g L^-1 ^and keeping this high concentration for the remainder of the production phase. This methanol concentration was very efficient for the intracellular production of HBsAg leading only to minor temporary growth arrest [35], however, these conditions were detrimental for the secretory production of IP. In this case, addition of methanol to final concentrations of 6 g L^-1 ^was leading to a drastical drop of the optical density concomitant to foam formation indicative of profound cell lysis (data not shown). Moreover, base consumption was replaced by acid consumption indicating the formation and/or release of alkaline compounds. Finally, cultivations had to be discontinued 10-15 h after methanol addition due to uncontrollable foaming.

**Figure 2:**
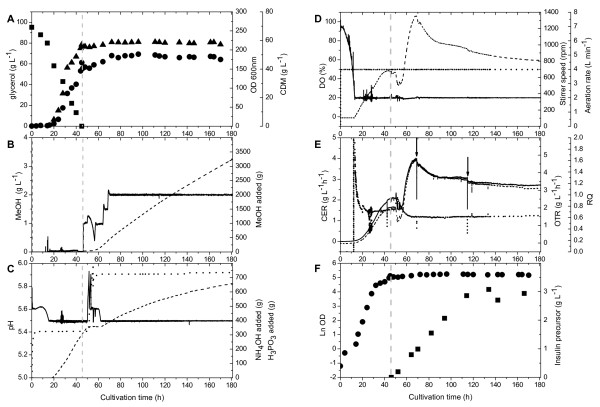
**Two-phase fed-batch cultivation of *P. pastoris *X-33 carrying the *AOX1 *promoter-driven IP gene**. Cells were first grown in a batch phase with glycerol as carbon source followed by a methanol feeding phase to induce the production of IP. (A) Concentrations of glycerol (filled squares) and biomass (optical density: filled circles; CDM: filled triangles). (B) Concentration of methanol (solid line) and amount of methanol added to the bioreactor (dashed line). (C) Medium pH (solid line), amount of ammonium hydroxide (dashed line), and amount of phosphoric acid added to the bioreactor (dotted line). (D) Dissolved oxygen concentration (solid line), aeration rate (dotted line), and stirrer speed (dashed line). (E) Oxygen transfer (dashed line) and carbon dioxide evolution (solid line) rates and respiratory quotient (dotted line). Arrows indicate removal of culture broth. (F) Cell growth (filled circles) and accumulation of IP (filled squares). The dashed vertical line indicates the end of the glycerol batch and the start of the methanol feeding phase.

Thus, we aimed at less drastic conditions to induce the secretory production of IP. After the depletion of glycerol (Fig. 2A), methanol was first added to a final concentration of 1 g L^-1 ^(Fig. 2B). With some time delay the cellular adjustment to methanol utilization was noticeable by a slight increase in the culture pH followed by acid addition for culture pH maintenance (Fig. 2C) and a decrease in the respiratory activity (Fig. 2D and 2E). Successful adaptation to methanol was then evident by the onset of methanol consumption (Fig. 2B) and the increase of the respiratory activity which started ~4 hours after methanol addition (Fig. 2D and 2E). About 20 and 24 h after the initial methanol pulse, the concentration of methanol in the bioreactor was further increased to 1.5 and 2 g L^-1^, respectively, and kept constant for the remainder of the cultivation without causing noteworthy process pertubations, e.g. uncontrollable foaming (Fig. 2B). The biomass concentration showed only a slight increase after the shift to methanol, increasing from 56 to 59 g L^-1 ^and starting to decline again after about 120 h growth on methanol (Fig. 2A).

**Figure 3:**
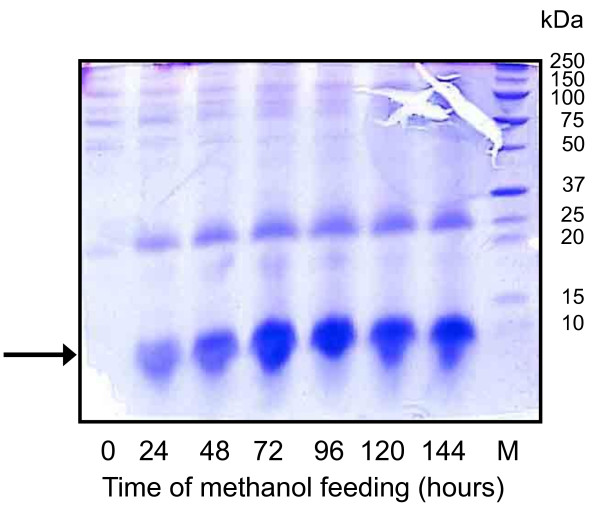
**Time course analysis of secretory IP production**. Cell-free culture supernatants were analyzed by 10% Tricine SDS-PAGE from samples taken directly before the addition of methanol (time 0) and 24, 48, 72, 96, 120 and 144 hours after the onset of methanol feeding. The arrow denotes the position of IP and M the lane of the molecular weight marker.

Recombinant IP production was analyzed by RP-HPLC and SDS-PAGE during the induction phase from culture aliquots withdrawn at various time points (Fig. 2F and 3, respectively). Extracellular IP concentrations increased during the first 80 h of growth on methanol reaching a maximum of 3.075 gram per liter of culture broth. Taking into account that the culture broth contained about 20% (v/v) biomass and 80% (v/v) cell free culture supernatant, the concentration of ~3 gram IP per liter culture broth translates into almost 4 gram IP per liter culture supernatant. Absence of excessive foaming and equal high product yields were also reached when the methanol concentration was immediately increased to a final concentration of 2 g L^-1 ^at the end of the glycerol batch phase (data not shown). Degradation of IP was not observed during the whole process. SDS-PAGE analysis of culture supernatants revealed that IP accumulated as the most prominent extracellular protein (Fig. 3).

### Purification of IP from the culture broth

Two different purification procedures were employed for the purification of IP from the culture broth. First, we aimed at the separation of the insulin precursor from the crude culture broth by using expanded bed absorption. This procedure omits the clarification of the culture broth by centrifugation and filtration, thus simplifying the down-stream process. To allow binding of the insulin precursor (theoretical p*I *4.74) to the resin in the expanded bed column, it was necessary to dilute and acidify the culture broth. After loading and washing the column in the expanded bed mode, the bound IP was eluted in normal mode using 1 mol L^-1 ^NaCl. The recovery of IP was not satisfactory, however, it could be increased two fold by supplementing the dilutent with the detergent Tween 20, which allowed more efficient binding and elution of IP from the resin. Under optimized conditions, ~1.5 g IP with a purity of 97% could be recovered from one liter culture broth, using expanded bed adsorption chromatography (Table 2).

**Table 2:** Purification of insulin precursor from microbial cultures

Expression host	Chromatography matrix	**Recovery (%) **^**a**^	**Purity (%) **^**a**^	**Reference **^**b**^
*E. coli *^c^	IgG-Sepharose	90	nd ^d^	[10]
*S. cerevisiae*	Streamline SP (XL) Expanded bed adsorption	88	nd	[43]
*P. pastoris*	XAD-7→ Sephadex-G50	>80 ^e^	60 ^e^	[26]
*P. pastoris*	CM-Sepharose FF	97	97	[27]
*P. pastoris*	Streamline SP (XL) Expanded bed adsorption	55	97	Present study
*P. pastoris*	IMAC-coupled with copper ions	95	96	Present study

^a^ Recovery and purity relates to the first chromatographic step

^b ^Only references on insulin precursor purification included containing respective quantitative data

^c ^Insulin precursor fused to IgG binding domains produced in the form of inclusion bodies

^d ^nd; not determined

^e ^Recovery and purity relates to the two-step purification procedure

**Figure 4:**
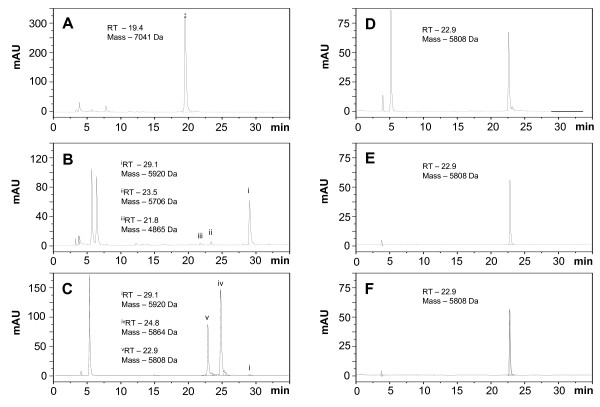
**RP-HPLC profiles of insulin species recovered after IMAC, transpeptidation and deprotection reactions, and final purification**. (A) RP-HPLC profile of the purified IP from IMAC. IP was eluted at a retention time of 19.4 minutes. (B) RP-HPLC profile of insulin species recovered after the transpeptidation reaction with i) pre-insulin H-Thr(tBu)-OtBu; ii) insulin species cleaved at B29 without the threonine ester; and iii) Insulin species cleaved at B22. (C) RP-HPLC profile of deprotection reaction in which the pre-insulin reaction mixture was incubated at room temperature for 5 minutes, with i) pre-insulin H-Thr(tBu)-OtBu; iv) insulin Thr-OtBu; and v) human insulin. (D) RP-HPLC profile of deprotection reaction in which the pre-insulin reaction mixture was incubated at room temperature for 60 minutes (E) RP-HPLC profile of purified human insulin. (F) RP-HPLC profile of mixture of human insulin and European Pharmacopoeia human insulin standard. The Phenomenex Jupiter C4 column was employed for quantification of insulin species. The identity of insulin species was determined by mass spectrometry as specified in the Materials and Methods section.

Furthermore, we wanted to explore if the natural metal binding affinities of human insulin and proinsulin [36-38] could be exploited for an immobilized metal affinity chromatography (IMAC) based purification step. Preliminary tests revealed that the insulin precursor can bind to IMAC columns precharged with Cu^2+ ^and Ni^2+ ^but - in contrast to insulin - not to Zn^2+^-precharged columns. Thus, the clarified culture broth was passed through Cu^2+ ^pre-charged chelating sepharose fast flow (GE Healthcare) and, after extensive washing to remove unbound impurities, the bound insulin precursor could be eluted at low pH. This step resulted in a recovery of 95% IP with a purity of 96% (Table 2, Fig. 4A).

### Conversion of IP to human insulin

The insulin precursor was transformed into pre-insulin by enzymatic transpeptidation in the presence of trypsin and O-t-butyl-L-threonine t-butyl ester (H-Thr(tBu)-OtBu). During this reaction, the N-terminal spacer peptide (EEAEAEAEPK) and the connecting linker peptide (AAK) are removed (Fig. 1A) and the threonine ester is added to B-29 in a transesterification reaction [33]. The efficiency of this reaction is around 90%. There are three species formed during the transpeptidation reaction which can be detected by analytical RP-HPLC (Fig. 4B). These species elute at retention times 21.8 min, 23.5 min, and 29.1 min and correspond to B22 cleaved IP, B29 cleaved IP without the threonine ester, and the desired reaction product pre-insulin-Thr(tBu)-OtBu (pre-insulin), respectively. B22 cleaved IP and B29 cleaved IP without the threonine ester represent only minor contaminants, the majority of IP is transformed into pre-insulin (Fig. 4B). Pre-insulin was purified from the transesterification mixture using semi-preparative gradient chromatography (Resource RPC column, Table 3).

**Table 3:** Purification of insulin precursor by IMAC and conversion to human insulin

Steps	Insulin species (molecular mass)	**Amount (mg) **^**a**^	**Amount (mmol) **^**a**^	**Recovery (%) **^**a**^	**Purity (%) **^**a**^
Culture broth ^b^	Insulin precursor 7041 Da	3075	0.437	100	20
Chelating sepharose FF ^c^	Insulin precursor 7041 Da	2921	0.415	95	96
Desalting & Lyophilization	Insulin precursor 7041 Da	2859	0.406	93	98
Transpeptidation	Pre-insulin 5920 Da	n.d. ^d^	n.d. ^d^	n.d. ^d^	n.d. ^d^
Resource RPC Desalting & Lyophilization	Pre-insulin 5920 Da	1915	0.323	74	90
Deprotection	Human insulin 5808 Da	n.d. ^d^	n.d. ^d^	n.d. ^d^	n.d. ^d^
Resource RPC (two passages), Desalting & Lyophilization	Human insulin 5808 Da	1537	0.264	60	99

^a ^Recovery and purity was determined by RP-HPLC

^b ^200 mg of IP were used for further processing. Data extrapolated to 1-L culture broth

^c ^Centrifugation step included before chromatography

^d ^n.d., not determined

The final recombinant human insulin was obtained from pre-insulin by removal of the blocking tertiary butyl groups from threonine in position B30 by acidolysis with trifluoroacetic acid (deprotection reaction). This step was tested at different temperatures (RT, ice, 10°C) and incubation times (from 30 min to 4 h). The highest yields were reached at RT with a reaction time of 60 min. After 60 min, more than 90% of pre-insulin is converted to human insulin (Fig. 4D). Only a minor fraction (~2%) of pre-insulin is converted into undesired by-products such as A21 desamido insulin. Finally, recombinant human insulin was purified by RP-HPLC as described in the Materials and Methods section. During this step, the A21 desamido insulin and other impurities are removed raising the purity of human insulin to 99% as determined by RP-HPLC (Fig. 4E) fully satisfying the European Pharmacopoeia requirements [39]. The total yield of the described process is around 60% (Table 3).

## Discussion

Production of recombinant proteins can be stressful to the host organism [40]. Compared to the intracellular production of HBsAg [35], the secretory production of IP is more harmful to the cells. Using high methanol concentrations for induction, extensive cell lysis is observed in the case of extracellular IP production but not in the case of the intracellular production of HBsAg. Although the extent of the cellular stress response is influenced by the nature of the recombinant product, membrane damage associated cell lysis is more likely to occur when the recombinant protein is directed towards the extracellular environment compared to intracellular product retention. In case of secretory protein production, less harsh induction conditions might be more advantageous to reduce growth perturbations to acceptable levels while keeping production levels high. For secretory IP production with *P. pastoris*, induction at a final methanol concentration of 2 g L^-1 ^is appropriate for high level production. This methanol concentration does not cause cell lysis and only leads to a neglectable increase in biomass during the production phase, thus, leading to optimal conditions for the recovery of proteins from the extracellular environment.

In conclusion, a simple two-phase cultivation process composed of a glycerol batch and a constant methanol fed-batch phase was adapted to secretory IP production. This approach increases IP levels more than 2 times compared to the highest previously reported value using *Pichia *based expression systems. Moreover, we propose a novel procedure to capture the insulin precursor from the culture supernatant using IMAC and, finally, show that ~1.5 g of 99% pure recombinant human insulin can be obtained per liter of culture broth.

## Materials and methods

### Strains and vectors

*P. pastoris *host strain X-33 and the plasmid vector pPICZα were purchased from Invitrogen. Plasmid pPICZα (Invitrogen Cat. No. V195-20) was used to construct the methanol inducible IP expression vector. A synthetic codon-optimized gene encoding the IP was inserted into the polylinker of this vector, in-frame with the α-factor secretion signal sequence to create the expression plasmid pPICZα-IP, which was then integrated into the genome of *P. pastoris *strain X-33. Transformation of *Pichia*, selection and screening of transformants to identify clones harboring the IP inserts, the isolation of putative multicopy clones using the zeocin screening protocol, and the identification of the methanol utilization (*mut*) phenotype were performed essentially as described previously [41].

### Culture conditions, control and *on-line *measurements

Precultures for high-cell density bioreactor cultivations were prepared as described before [35]. High-cell density cultivations were carried out in a 15-L BIOSTAT-C (B. Braun Biotech International, Germany) bioreactor essentially as described previously [35] with minor modifications. A 1-L preculture was transferred to the bioreactor containing 7 L growth medium. The growth medium contained per liter: glycerol, 95.2 g; potassium *di*-hydrogen phosphate, 9.4 g; yeast trace metal (YTM) solution, 4.56 g; ammonium sulfate, 15.7 g; magnesium sulfate *hepta*-hydrate, 4.6 g; calcium chloride *di*-hydrate, 0.28 g; and biotin, 0.4 mg. The YTM solution contained: potassium iodide, 207.5 mg L^-1^; manganese sulfate, 760.6 mg L^-1^; *di*-sodium molybdate, 484 mg L^-1^; boric acid, 46.3 mg L^-1^; zinc sulfate *hepta*-hydrate, 5.032 g L^-1^; ferric chloride *hexa*-hydrate, 12.0 g L^-1^; and sulfuric acid, 9.2 g L^-1^. Foaming was controlled by the addition of antifoam (Ucolub N115). Temperature was maintained at 30°C and pH at pH 5.5 with 12.5% (v/v) NH_4_OH or 1 mol L^-1 ^H_3_PO_4_. Aeration rate was maintained at 4 L min^-1 ^throughout the process. The stirrer speed was controlled between 100 to 1370 rpm aiming at a dissolved oxygen (DO) concentration of 20% air saturation. After consumption of glycerol, indicated by an increase of the DO concentration, production of recombinant IP was initiated by step-wise addition of a methanol solution [96.6% (w/w) methanol and 4.4% (w/w) YTM] to a final methanol concentration of 2 g L^-1^, which was maintained constant throughout the remainder of the induction period based on *on-line *measured methanol concentrations determined from the methanol vapor in the off-gas using a flame ionization detector (Ratfish Instruments, Germany). Based on the gas liquid phase equilibrium methanol concentrations were determined in the off-gas using two point calibrations directly before and after induction. The concentrations of oxygen and carbon dioxide in the exhaust gas were determined by paramagnetic and infrared exhaust gas analysis systems, respectively (Maihak, Hamburg, Germany).

### Determination of cell concentration

The cell concentration of suitably diluted culture samples was measured by optical density (OD) at 600 nm using a Novaspec II spectrophotometer (Pharmacia LKB). For cell dry mass (CDM) determination, 1-mL aliquots of the culture broth were pelleted (13,000 rpm for 15 min at room temperature using an Eppendorf microcentrifuge, model 5415C) in pre-weighed tubes, re-suspended in 50 mmol L^-1 ^phosphate buffer (pH 7.2), re-centrifuged and the resultant pellets vacuum-dried at 80°C (Heraeus Instruments, Vacutherm) to constant mass. CDM was measured in triplicates and averaged.

### Determination of glycerol concentration

Culture samples (1-mL) were centrifuged (13,000 rpm for 15 min at 4°C) and the glycerol concentration in the supernatant analyzed in duplicate using glycerol test kits (Roche, Basel, Switzerland).

### Protein analysis

Clarified culture broth samples were analyzed by denaturing 10%-polyacrylamide gel electrophoresis using the Tricine buffer system [42]. Samples (40-μL) were mixed with an equal volume of loading buffer [250 mmol L^-1 ^Tricine (pH 8.5), 10% β-mercaptoethanol, 2% SDS, 1% glycerol, and 0.1% bromophenol blue], vortexed for 1 min and boiled for 15 min, vortexed again, clarified by centrifugation (max speed, 15 min, RT) and loaded on the gel (20 μL per lane). Following electrophoresis, the separated polypeptides were visualized using Coomassie blue staining.

Reversed-phase HPLC (RP-HPLC) was used for quantification of IP from culture broth. Filtered aliquots of *Pichia *culture broth (200-μL) were mixed with an equal volume of solution A [0.15% (v/v) TFA in MilliQ water] and analyzed by RP-HPLC using a 3 μm SUPELCOSIL™ LC-304 column (3.3 cm × 4.6 mm) with a Schimadzu liquid chromatography system, equipped with an auto-injector (SIL-10AD *VP*), UV-VIS detector (SPD-10A), pumps A and B (LC-10AT *VP*), and a controller (SCL-10A *VP*). The HPLC column was maintained at 24°C (HPLC column oven, CH-500, Eppendorf, Germany). Elution was done with a gradient formed by mixing solutions A and B [0.15% (v/v) TFA in acetonitrile] as follows: 10% B (0 - 6 min), 10 - 43% B (6 - 41 min), 43 - 100% B (41 - 43 min), 100 - 10% B (43 - 53 min), 10% B (53 - 60 min). The flow rate was maintained at 1 mL min^-1^. The column effluent was monitored at 280 nm.

Reversed-phase HPLC (RP-HPLC) was also used for characterization and quantification of IP and insulin species after purifications, transpeptidation, and deprotection. These samples were loaded on a 5 μm Phenomenex Jupiter C4 column (300A, 250 mm × 4.6 mm) in solution A [0.1% (v/v) TFA in MilliQ water]. Elution was done at 25°C with a gradient formed by mixing solutions A and B [0.1% (v/v) TFA in acetonitrile] as follows: 0 - 20% B (0 - 6 min), 20 - 55% B (6 - 41 min), 55 - 100% B (41 - 42 min). The flow rate was also 1 mL min^-1 ^and the column effluent was monitored at 280 nm.

### Purification of IP using expanded bed absorption

The culture broth (1-L) was diluted with 4 liter of 1% Tween 20 (pH 2.0, 4.7 mS cm^-1^) to reduce the salt content (conductivity of culture broth was 24.2 mS cm^-1^), the pH and the viscosity for more efficient binding to the Streamline SP XL resin (Amersham Pharmacia Biotech, Sweden). A Streamline 50 column (Amersham Pharmacia Biotech, Sweden) packed with Streamline SP XL resin to a sedimented height of 13 cm and pre-equilibrated with 1% Tween 20 (pH 2.0; 4.7 mS cm^-1^) was employed and the diluted and well mixed culture broth was pumped from the lower end of the column leading to an expansion of the sedimented height to 24.4 cm (*H/H_o _*expansion ratio = 1.88, where *H *and *H_o _*represent the heights of the column bed after and before expansion, respectively). After loading, the column was washed with MilliQ water (pH 5.8) until the absorbance at 280 nm returned to base line in the eluate. Following, the bound IP was eluted using conventional chromatography by reversing the flow in a now settled bed using 1 mol L^-1 ^NaCl (pH 7.5, conductivity ~100 mS cm^-1^). Protein containing fractions (absorbance at 280 nm) were pooled and loaded on a preparative C18 bulk column (125 A^0 ^55-105 μm, Waters Corporation, Milford, MA, USA) pre-equilibrated with 10% acetonitrile and 0.1% TFA. After loading, the column was washed with 10% acetonitrile and 0.1% TFA and elution was carried out with 60% acetonitrile and 0.1% TFA. The eluted protein was lyophilized prior to further treatment (Labconco freeze dry system, Germany).

### Purification of IP using íon metal affinity chromatography

The culture broth (1-L) was centrifuged (Sorvall RC5BPlus centrifuge, SLA 3000 rotor) at 4°C and 3,000 rpm for 30 min. The supernatant was filtered using a 0.22 μm filter, the pH adjusted to pH 7.0 and the clarified culture supernatant allowed to bind to Cu^2+ ^pre-charged chelating sepharose fast flow (GE Healthcare) pre-equilibrated in equilibration buffer [20 mmol L^-1 ^sodium phosphate buffer (pH 7.0), 500 mmol L^-1 ^NaCl] at room temperature. A maximum of 200 mg IP was used per 50 mL column volume. After sample loading, the column was extensively washed with equilibration buffer to remove unbound proteins. The bound IP was eluted by a linear pH gradient using a low pH elution buffer [20 mmol L^-1 ^sodium phosphate buffer (pH 3.15), 500 mmol L^-1 ^NaCl]. The purified IP was diluted with MilliQ water to lower the conductivity to appr. 8 mS cm^-1 ^and the pH adjusted to pH 4.0 to allow binding to HiTrap SP HP column (GE Healthcare). Elution was performed using 100 mmol L^-1 ^NH_4_HCO_3 _(pH 9.0). The purified IP was lyophilized prior to the conversion to pre-insulin by transpeptidation.

### Transpeptidation, deprotection and final purification of human insulin

The transpeptidation reaction was essentially performed as described previously [33] with minor modifications. Briefly, the reaction mixture contained 10 mmol L^-1 ^insulin precursor, 200 μmol L^-1 ^trypsin, 0.8 mol L^-1 ^H-Thr(tBu)-OtBu, 7 mmol L^-1 ^CaCl_2_, 50% dimethylformamide/ethanol 1:1 (v/v) and 26% water with the pH adjusted to pH 6.0 with acetic acid. The reaction was carried out at 12°C for 24 hrs. The reaction mixture was subjected to semi-preparative gradient chromatography for purification of pre-insulin using a Resource RPC column (GE Healthcare). The bound pre-insulin was eluted by a gradient formed by mixing solutions A (0.2 mol L^-1 ^ammonium sulphate buffer, pH 2.0) and B (0.1 mol L^-1 ^ammonium sulphate buffer with 40% (v/v) acetonitrile) as follows: 0 - 45% B (16.6 column volumes, CV), 45% B (16.6 CV), 45 - 75% B (16.6 CV), 75% B (2.7 CV), 75 - 100% B (3 CV). The purified pre-insulin was desalted by cation exchange chromatography (HiTrap SP HP column, GE Healthcare, elution with 100 mmol L^-1 ^NH_4_HCO_3_, pH 9) and lyophilized prior to further processing.

Pre-insulin was transformed to human insulin by removal of the tertiary-butyl groups of threonine in position B30 by acidolysis using trifluoroacetic acid (19 μL TFA per mg pre-insulin). To protect the polypeptide from degradation and desamidation, 0.05 mg each of L-tryptophan and L-glutamine per mg pre-insulin were included as scavengers in the reaction mixture which also contained 2.5% water.

Final purification of human insulin was carried out by semi-preparative gradient chromatography (Resource RPC column, GE Healthcare). The deprotection reaction mixture was subjected to gradient chromatography by mixing solutions A [90% ammonium acetate buffer (1 mol L^-1^, pH 7.0) and 10% acetonitrile] and B [50% ammonium acetate buffer (1 mol L^-1^, pH 7.0) and 50% acetonitrile] as follows: 0 - 35% B (1 CV), 35% B (26.6 CV), 35 - 50% B (6 CV), 50% B (6 CV), 50 - 100% B (4 CV). The eluted recombinant human insulin was desalted and lyophilized as described above for long term storage.

### Mass spectrometry

Electrospray mass spectrometry (ESI-MS) was performed with API 150EX, PE SCIEX instrument (Applied Biosystems). Samples eluted from Resource RPC columns were desalted (HiTrap SP HP column, GE Healthcare) and lyophilized. Lyophilized samples were reconstituted in 0.1% TFA and 80% acetonitrile in MilliQ water and analyzed by direct infusion (5 μL min^-1^) in the interval of 600-2500 m/z. Spectra were acquired using LC2Tune 1.4 Software and the molecular mass was obtained by automatic deconvolution using the Biomultiview software.

## Competing interests

The authors declare that they have no competing interests.

## Authors' contributions

CG and AA carried out the cultivations. CG and SP carried out the purification. CG performed the IP determinations. TG implemented the methanol controller. NS and ST were responsible for the design of the IP clone, purification of IP and transpeptidation. DC and NK were responsible for the generation of the *Pichia pastoris *clone. SS and NK prepared the initial draft. UR conceived and directed the cultivation experiments and prepared the final manuscript. All authors read and approved the final manuscript.
